# Waste *Citrus limon* Leaves as Source of Essential Oil Rich in Limonene and Citral: Chemical Characterization, Antimicrobial and Antioxidant Properties, and Effects on Cancer Cell Viability

**DOI:** 10.3390/antiox12061238

**Published:** 2023-06-08

**Authors:** Giacomo Luigi Petretto, Giuseppe Vacca, Roberta Addis, Giorgio Pintore, Mariella Nieddu, Franca Piras, Valeria Sogos, Francesco Fancello, Severino Zara, Antonella Rosa

**Affiliations:** 1 Department of Medicine, Surgery and Pharmacy, University of Sassari, Viale San Pietro, 07100 Sassari, Italy; gpetretto@uniss.it (G.L.P.); g.vacca4@studenti.uniss.it (G.V.); raddis@uniss.it (R.A.); pintore@uniss.it (G.P.); 2 Department of Biomedical Sciences, University of Cagliari, Cittadella Universitaria, 09042 Monserrato, CA, Italy; mnieddu@unica.it (M.N.); fpiras@unica.it (F.P.); sogos@unica.it (V.S.); 3 Department of Agriculture, University of Sassari, Viale Italia, 07100 Sassari, Italy; fancello@uniss.it (F.F.); szara@uniss.it (S.Z.)

**Keywords:** *Citrus limon*, discarded leaves, leaf essential oil, bioactivity, cytotoxicity

## Abstract

This study investigated chemical composition, cytotoxicity in normal and cancer cells, and antimicrobial and antioxidant activity of the essential oil (EO) isolated by hydrodistillation from the discarded leaves of lemon (*Citrus limon*) plants cultivated in Sardinia (Italy). The volatile chemical composition of lemon leaf EO (LLEO) was analyzed with gas chromatography-mass spectrometry combined with flame ionization detection (GC/MS and GC/FID). The most abundant component of LLEO was limonene (260.7 mg/mL), followed by geranial (102.6 mg/mL) and neral (88.3 mg/mL). The antimicrobial activity of LLEO was tested using eight bacterial strains and two types of yeasts by a microdilution broth test. *Candida albicans* showed the greatest susceptibility (MIC = 0.625 μL/mL) and *Listeria monocytogenes* and *Staphylococcus aureus* were inhibited at low LLEO concentration (MIC values from 2.5 to 5 μL/mL). The *C. limon* leaf EO displayed radical scavenging ability (IC_50_ value of 10.24 mg/mL) in the 2,2-diphenyl-1-picryl-hydrazylhydrate (DPPH) assay. Furthermore, the LLEO impact on cell viability was explored by 3-(4,5-dimethylthiazol-2-yl)-2,5-diphenyltetrazolium bromide (MTT) assay in cancer HeLa cells, A375 melanoma cell line, normal fibroblasts (3T3 cells), and keratinocytes (HaCaT cells). LLEO, at 24 h of incubation, significantly reduced viability from 25 μM in Hela cells (33% reduction) and A375 cells (27%), greatly affecting cell morphology, whereas this effect was found from 50 μM on 3T3 fibroblasts and keratinocytes. LLEO’s pro-oxidant effect was also established in HeLa cells by 2′,7′-dichlorodihydrofluorescein diacetate assay.

## 1. Introduction

Citrus plants (Rutaceae family) represent one of the most important fruit crops cultivated in the world [1,2], particularly in the Mediterranean basin area [3]. Citrus species (orange, lemon, mandarin, grapefruit, clementine, and bergamot) are largely cultivated in Italy for alimentary or industrial purposes (fresh fruit consumption, beverages, cosmetics, sweets, and pharmaceuticals) [3,4,5]. Moreover, Citrus fruit by-products (pulp residue, albedo, peels, and seeds) are a source of bioactive compounds with potential for manufactured foods, health care, and animal feed [3,4,5,6,7].

Citrus plants constitute one of the main valuable sources of essential oils (EOs) in the world [2,3,8,9]. Citrus EOs, primarily extracted from the peels [3,4,8,9] but also from leaves [9,10,11], flowers [1,9], young shoot [3], buds [3,9], seeds, and roots [9], are aromatic volatile liquids, easily extracted by steam distillation [3,9]. Citrus EOs are very complex mixtures of organic components with monoterpenes and sesquiterpenes as well as their oxygenated derivatives, aliphatic aldehydes, alcohols, and esters constituting the major fractions, and are amply used for their numerous biological activities [1,2,3,4,5,8,9,10,11,12].

One of the best-known and most used species of the genus Citrus is the lemon (*Citrus limon*) [3,13]. *C. limon*, like many other prolific citrus species, gives rise to numerous varieties, cultivars, and hybrids [3,13]. Lemon fruits are well-known for their valuable nutritional, pharmaceutical, and cosmetic properties [3,4,5,13]. The main raw material of *C. limon* is the fruit, particularly the juice and EO obtained from it [3,4,5,13]. In Italy, the albedo and the external part (flavedo or epicarp) of *C. limon* fruits are largely used to produce sweets in regional traditional pastries [4,5]. The flavedo is also used for the preparation of liqueurs by hydroalcoholic maceration [14]. In addition to pericarp and fruit, the European Food Safety Authority (EFSA) classified leaves of *C. limon* as raw materials of plant origin, in which there is the presence of naturally occurring ingredients that may pose a threat to human health when used in the production of food and dietary supplements [13].

Lemon EOs, obtained by cold pressing of the peel or distillation of leaves, are broadly applied as an aroma enhancer in beverages, bakery, and food products, as a flavoring agent in pharmaceutical preparations, and as a fragrance in perfumery/cosmetic industries [13,15]. Lemon leaves (evergreen and lanceolate) represent an important source of EO, which ranges approximately between 0.33–0.34% [10,16] and 0.56% [17] (by weight) and varies greatly with the variety and/or cultivars, the geographic origin, and the harvesting period [3,13,15]. The EO of the *C. limon* leaves differs in composition from oil obtained from pericarp in which limonene generally represents the major component [4,13,18]. Whereas the *C. limon* leaf EO is generally rich in limonene [11,13,18], in some cases, other compounds have been identified as the major constituents [10,15,16,17,19]. Other reported main compounds include neral, geranial, sabinene, citronellal, linalool, (E)-β-ocimene, geranyl acetate, geraniol, alpha-terpineol, linalyl acetate, and myrcene [10,11,13,15,16,17,18,19]; however, significant quali-quantitative differences in *C. limon* leaf EO chemical composition have emerged among data in the literature [10,11,13,15,16,17,18,19].

Several studies have been performed on analgesic [9], antimicrobial [11,15,16,17,19,20], anti-leishmanial [10], insecticidal [16], and antioxidant activities [11,15,18] of the EOs obtained from the leaves of various species and/or cultivars of lemon around the world. Moreover, *C. limon* EOs and their aroma components (limonene, linalyl acetate, geranial, and neral) have drawn the attention of researchers for their anticancer activity [9,21,22,23,24,25,26]; however, limited research has been specifically conducted on the antitumor properties and cytotoxicity of lemon leaf EO [27,28]. Recent studies of eukaryotic cells have demonstrated that EOs exert pro-oxidant and cytotoxic effects [29]. Therefore, for the effective use of EOs as food preservatives and for clinical application, an evaluation of their cytotoxicity and the identification of the mechanisms affecting cell viability are required [29].

The island of Sardinia is one of the major producers of *Citrus* fruits in Italy [4] and lemon represents about 14% of the most produced *Citrus* fruits in this region. A great number of leaves, which are produced by the pruning [18] of lemon trees, are often discarded as an agro-industrial waste product, with some adverse effects on the local environment and ecology. Therefore, the utilization of discarded lemon leaves can significantly decrease the problems of their final disposal and increase the added value of the agricultural process [15,20].

**Figure 1:**
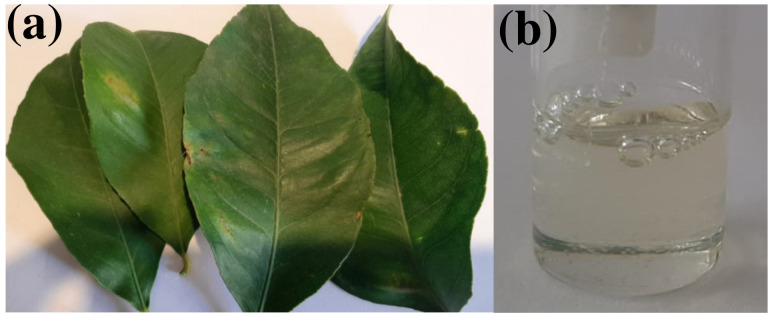
Digital image of *Citrus limon* leaves (**a**) and essential oil (LLEO) obtained by steam distillation of the leaves (**b**).

To our knowledge, reports that have comprehensively examined the pleiotropic biological effects of the EO derived from the leaves of *C. limon* plants in relation to the chemical constituents are still lacking. The present work aimed to investigate the chemical composition and the biological properties (antioxidant, antimicrobial, and cytotoxic profile) of the EO (Figure 1) obtained by hydrodistillation from leaves of lemon discarded by a local farm (Sardinia, Italy).

The volatile component profile of lemon leaf EO (LLEO) was determined by chromatographic techniques (GC-FID and GC-MS). The antimicrobial activity of LLEO was tested using eight bacterial strains and two types of yeast by a microdilution broth test. Then, the antioxidant properties of LLEO were evaluated by 2,2-diphenylpicrylhydrazyl (DPPH) assay. Moreover, the LLEO effect (24 h of incubation) on cell viability was tested in cancer cell lines (human cervical cancer HeLa cells and human melanoma A375 cells) and normal cells (murine 3T3 fibroblasts and human HaCaT keratinocytes), together with the investigation of the changes occurring in the cell morphology. Finally, the intracellular generation of reactive oxygen species (ROS) was explored in cancer HeLa cells during LLEO treatment for 2 h. The relation between the chemical composition, the observed bioactivity, and the main mechanism of action was explored.

## 2. Materials and Methods

### 2.1. Chemicals and Reagents

Dimethyl sulfoxide (DMSO), hydrogen peroxide (H_2_O_2_) solution 30% (*w*/*w*), and 2′,7′-dichlorodihydrofluorescein diacetate (H_2_-DCF-DA) were purchased from Merck Life Science (Milan, Italy). Citral and 3-(4,5-dimethylthiazol-2-yl)-2,5-diphenyltetrazolium bromide (MTT) were purchased from Sigma–Aldrich (Milan, Italy). Limonene and caryophyllene were purchased from Fluka AG (Steinheim, Germany). Solvents used for the analysis were acquired from Carlo Erba Reagenti (Milan, Italy). Cell culture material was supplied by Invitrogen and EuroClone (Milan, Italy). All the chemicals used in this study were of analytical grade.

### 2.2. Plant Material and LEO Extraction

Lemon leaves (11.0 kg) were collected in June 2022 at the “Tirso Agrumi” farm from five adult plants (after pruning) regularly irrigated and cultivated in the Tirso river valley near Solarussa (OR, Sardinia, Italy). The leaf samples were kept at −18 °C after collection until extraction. Samples of lemon leaves (2.2 kg), coarsely grounded, were suspended in 3 L of water and subjected to hydrodistillation using a Clevenger-type apparatus for 2 h as previously reported [11]. Five repeated-batch cycles of extraction were carried out and the obtained LLEO was collected separately, dried over anhydrous sodium sulfate (Na_2_SO_4_), and then stored under a nitrogen atmosphere at 4 °C in amber glass vials until analysis.

### 2.3. Gas Chromatograph-Mass Spectrometer (GC-MS) Analysis

The GC-MS analysis of the LLEO in hexane (1:100 dilution) was carried out using an Agilent 6850 GC system coupled with an Agilent 5973 Mass Selective Detector. The chromatographic separation was performed on an HP-5 capillary column (30 m × 0.25 mm, film thickness 0.17 μm). The following temperature program was used: 50 °C was held for 3 min, then increased to 210 °C at a rate of 4 °C/min, held for 15 min, and then increased at a rate of 10 °C/min up to 300 °C, which was finally maintained for 15 min. Helium was used as the carrier gas at a constant flow rate of 1 mL/min. Individual identification of the components was carried out by comparing the fragmentation spectra of the unknown molecules, separated into peaks by the GC component, with the spectra of known molecules available on the NIST online database. By comparing the Retention Indices of the unknown components of the EO that were experimentally detected with those of the NIST database, it was possible to further confirm the identification of the single molecules characterized by the fragmentation spectrum obtained by GC-MS. A solution of linear alkanes (C_7_–C_22_) was initially prepared and analyzed according to the same instrumental program applied for the EO samples in the same chromatographic column (HP-5) and Van den Dool and Kartz’s equation was applied in the calculation of the retention index (RI) [30].

### 2.4. Gas Chromatograph Analysis with Flame Ionization Detector (GC-FID)

The GC-FID analysis of the LLEO in hexane (1:100 dilution) was carried out using an Agilent 4890 GC with an HP-5 capillary column (30 m × 0.25 mm, film thickness 0.17 μm). Helium was used as carrier gas at a constant flow rate of 1 mL/min. The temperature program was similar to the one already applied for GC-MS analysis. To quantify the concentration of the LLEO components, a stock solution in hexane of commercially available standards for each class of compound (hydrocarbon monoterpenes, oxygenated monoterpenes, hydrocarbon sesquiterpenes) was composed according to the literature [31] with known amounts of dodecane as an internal standard to implement the calibration. The standards used were limonene (as a reference for hydrocarbon monoterpenes; 3 mg/mL), citral (neral and geranial, as a reference for oxygenated monoterpenoids; 5.1 mg/mL), caryophyllene (as a reference for hydrocarbon sesquiterpenes; 0.65 mg/mL).

The linearity range was evaluated by injecting serial dilutions of the stock solution (from 5% to 30%) into the GC-FID, and the calibration lines were obtained using the ratios between the areas of the peaks of the molecules of interest and the internal standard and the concentration (expressed in ppm). Subsequently, the LLEO diluted in hexane in concentrations from 1 to 3% *v*/*v* was analyzed with a fixed concentration of dodecane as an internal standard under the same operating conditions. The concentration values of the single molecules in the distillation product were extrapolated from the calibration lines, obtained by analyzing the dilutions of the stock solution, and from the ratios between the internal standard and the peak areas of the components in the LLEO.

### 2.5. Antimicrobial Analysis

The minimum inhibitory concentration (MIC) of the LLEO was tested on two types of yeast (*Candida albicans* 3933 and *Candida albicans* 3993) and eight types of bacteria (*Staphylococcus aureus* DSM 20231, *S. aureus* DSM 2569, *S. aureus* DSM 6148, *Listeria monocytogenes* DSM 20600, *L. monocytogenes* DSM 15675, *Escherichia coli* DSM 30083, *E. coli* DSM 4415, and *Salmonella bongori* DSM 13772) species through the culture broth microdilution method according to the M07-A9 standard [32], as previously reported [11]. Stock solutions of LLEO were prepared with a concentration of 40 μL/mL and subsequently diluted in Muller Hinton agar for bacteria and in YEPD (2% Yeast Extract, 1% Peptone, 2% Dextrose) for yeasts to obtain a range of concentrations from 0.078 to 20 μL/mL. Aliquots (100 μL) of inoculation of the desired dilution were added to a 96-well microdilution plate, in which 100 µL of LLEO at the desired dilution was already placed in each well. The plate was incubated at 37 °C for 24 h. After the incubation, the MICs (μL/mL) were determined, defined as the lowest concentration of EO capable of inhibiting the growth of the microorganisms under examination, indicated by the absence of turbidity in the solution. The minimal microbicidal concentrations (MMCs) were also determined by streak 50 μL of well suspension that did not show visible growth of microorganisms in Petri dishes containing agarized medium used for the determination of the MIC. DMSO (dimethyl sulfoxide) at 1% concentration was used as a negative control; each assay was performed in quadruplicate and the experiments were repeated twice.

### 2.6. DPPH Assay

Different LLEO concentrations, ranging from 0.5 up to a maximum of 5 mg, were added to a 2,2-diphenyl-1-picrylhydrazyl (DPPH) solution (100 μM in ethyl acetate) to reach a final volume of 1 mL. The DPPH^●^ free radical scavenging activity of LLEO was determined according to a previously described method [33]. Briefly, the mixtures were shaken and incubated in the dark for 50 min, and the OD values were read at 517 nm. A Trolox calibration curve in the range of 0.25–7.5 μg/mL was used as the positive reference. The percentage of free radical scavenging activities was calculated as follows:

DPPH scavenging activity (%) = [(A_blank_ − A_sample_)/A_blank_] × 100

where A_blank_ is the absorbance of the control reaction (containing all reagents except the test compound), and A_sample_ is the absorbance in the presence of LLEO.

### 2.7. Cell Cultures

HeLa cell line, derived from a human cervical epithelioid carcinoma, A375 human melanoma cell line, and mouse 3T3 fibroblasts were obtained from the American Type Culture Collection (ATCC, Rockville, MD, USA). HaCaT cell line (human keratinocyte cells) was obtained by CLS-Cell Line Services (Eppelheim, Germany). All cell lines were grown in Dulbecco’s modified Eagle’s medium (DMEM) with high glucose, supplemented with 2 mM L-glutamine, penicillin (100 units/mL)–streptomycin (100 μg/mL), and fetal calf serum (FCS) (10% *v*/*v*), at 37 °C in a 5% CO_2_ incubator. Subcultures of all cell types were grown in T-75 culture flasks and passaged with a trypsin-EDTA solution.

### 2.8. Cytotoxic Activity (MTT Assay) in Cancer and Normal Cells

The cytotoxic effect of LLEO was evaluated in cancer HeLa cells, A375 melanoma cells, 3T3 fibroblasts, and HaCaT cells by the MTT colorimetric assay [34,35]. Cells were seeded in 96-well plates at a density of 3 × 10^4^ cells/mL (HeLa and A375 cells), 10^5^ cells/mL (HaCaT cells), and 3 × 10^5^ cells/mL (3T3 fibroblasts) in 100 μL of complete culture medium and cultured for 48 h. Cells (at 80–90% cell confluence) were subsequently incubated (24 h) with various concentrations (2.5–500 μg/mL) of LLEO (from 50 mg/mL and 5 mg/mL solutions in DMSO) in a fresh medium (treated cells). Treated cells were compared for viability to control cells (non-treated) and cells (vehicle-treated cells) incubated for 24 h with an equivalent volume of DMSO (maximal final concentration, 1%). After incubation, cells were subjected to the MTT viability test as previously reported [34,35]. Color development (absorbance proportional to the number of viable cells) was measured at 570 nm with an Infinite 200 auto microplate reader (Infinite 200, Tecan, Austria) and results were expressed as a percentage of cell viability in comparison with control cells. Preliminary evaluation of the cell morphology after 24 h of incubation with various amounts (2.5–500 μg/mL) of LLEO was performed by microscopic analysis with a ZOE™ Fluorescent Cell Imager (Bio-Rad Laboratories, Inc., Hercules, CA, USA).

### 2.9. ROS Production in Cancer Cells

Changes in the mitochondrial redox status of cancer HeLa cells in response to LLEO were determined. Intracellular ROS production was monitored in cells by adding the 2’,7’-dichlorodihydrofluorescein diacetate (H_2_-DCF-DA), a fluorogenic biosensor, as previously reported [4,36] with some modification. Briefly, HeLa cells were seeded in 96-well plates at a density of 3 × 10^4^ cells/mL in 100 μL of medium and cultured for 48 h. Cells (at 70% confluence) were subsequently incubated in phosphate-buffered saline (PBS) solution (pH 7.4) with 10 μM H_2_-DCF-DA for 30 min. After incubation, the PBS with H_2_-DCF-DA was removed, and the cells were washed before the addition of fresh medium alone (control) or with different LLEO concentrations (2.5–500 μg/mL, from a 50 mg/mL solution in DMSO). Increases in cell fluorescence were measured (every 5 min) for 2 h at excitation and emission wavelengths of 490 and 520 nm, respectively, using an Infinite 200 auto microplate reader, maintaining the temperature at 25 °C. Data were collected and analyzed using the Tecan I-control 1.5 V software. This method provides a direct measure of overall oxidative stress [4,36], which detects intracellular oxidants. H_2_-DCF-DA is taken up by the cells and then deacetylated by intracellular esterases. The resulting H_2_-DCF becomes trapped inside the cell, and the oxidation of the non-fluorescent 2,7-dichlorodihydrofluorescein moiety H_2_-DCF by ROS to the highly fluorescent DCF is possible [36]. Evaluation of the cell morphology at the end of cell fluorescence measurement was also performed by microscopic analysis with a ZOE™ Fluorescent Cell Imager.

### 2.10. Effect Versus H_2_O_2_-Induced Oxidation in HaCaT Cells

The H_2_-DCF-DA assay was also used to monitor the LLEO effect in HaCaT cells against the intracellular ROS production induced by H_2_O_2_. Briefly, HaCaT cells were seeded in 96-well plates at a density of 10^5^ cells/mL in 100 μL of medium and cultured for 48 h. Cells (at 90% confluence) were subsequently incubated in a fresh medium for 24 h with a non-cytotoxic LLEO concentration (10 μg/mL, from a 50 mg/mL solution in DMSO). After treatment, cells were washed with PBS and then incubated in PBS with 10 μM H_2_-DCF-DA for 30 min. After incubation, cells were washed before the addition of PBS alone (control) or PBS with the oxidant compound H_2_O_2_ (0.5, 1, and 2.5 mM). The increases in cell fluorescence were measured for 1 h, as reported above in Section 2.9.

### 2.11. Statistical Analysis

Evaluation of the statistical significance of differences was performed using Graph Pad INSTAT software (GraphPad Software, San Diego, CA, USA). Results were expressed as mean ± standard deviation (SD), and statistically significant differences were evaluated with *p* < 0.05 as a minimal level of significance. Multiple comparison of means groups was assessed by one-way analysis of variance (One-way ANOVA) followed by the Bonferroni Multiple Comparisons Test to substantiate statistical differences between groups.

## 3. Results

### 3.1. Chemical Composition

The hydrodistillation of discarded *C. limon* leaves gave a slight yellow EO with a pleasant herbaceous smell with a yield of 2.035% (*v*/*w*) calculated from the dry weight. The obtained EO was then subjected to both analytical protocols and chemical–biological study. The chemical analyses were focused on both qualitative and quantitative data. The GC-MS technique was used to reveal the LLEO chemical composition, whereas the GC-FID technique was applied to obtain quantitative data.

**Figure 2:**
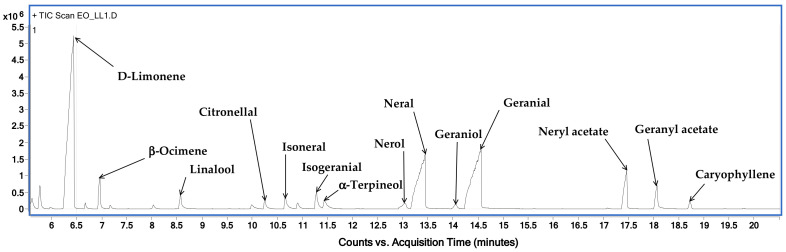
GC-MS chromatogram, obtained on an HP-5 capillary column, of *C. limon* leaf essential oil (LLEO).

Figure 2 shows the chromatographic profile of LLEO by GC–MS techniques (on a HP-5 capillary column) with the indication of the main identified volatile compounds, while the LLEO chemical composition, expressed as mg for mL, is reported in Table 1.

GC-MS analysis revealed the presence of 21 compounds and among them, limonene was found to be the major component, accounting for 256.7 ± 2.4 mg/mL, followed by citral at a concentration of 194.5 mg/mL, as the sum of oxygenated monoterpenes geranial (106.2 ± 1.6 mg/mL) and neral (88.3 ± 1.3 mg/mL). LLEO was also characterized by high amounts of myrcene (94.7 ± 1.9 mg/mL), neryl acetate (31.8 ± 0.9 mg/mL), linalool (24.5 ± 0.3 mg/mL), geranyl acetate (23.5 ± 0.9 mg/mL), and 3-carene (20.6 ± 1.2 mg/mL). Other components, with relatively small amounts, were beta-ocimene, citronellal, alpha-terpineol, nerol, and terpinene-4-ol.

### 3.2. Antimicrobial Activity

In this work, the antimicrobial activity of LLEO was tested through the culture broth microdilution method against foodborne pathogenic microorganisms, two yeast (*Candida albicans* 3933 and *Candida albicans* 3993) and eight bacterial (*Staphylococcus aureus* DSM 20231, *S. aureus* DSM 2569, *S. aureus* DSM 6148, *Listeria monocytogenes* DSM 20600, *L. monocytogenes* DSM 15675, *Escherichia coli* DSM 30083, *E. coli* DSM 4415, and *Salmonella bongori* DSM 13772) species. Foods contaminated with *L. monocytogenes*, *S. aureus*, and *E. coli* have been reported as the causal agents of foodborne diseases [1].

The minimum inhibitory concentrations (MICs) and minimum microbicidal concentrations (MMCs) expressed as μl/mL, of LLEO against the tested microorganisms after 24 h of incubation are reported in Table 2.

The two yeast strains of *C. albicans* showed the greatest susceptibility as their proliferation was inhibited following exposure to low LLEO concentrations (MIC = 0.625 μL/mL). Even the MMC is twice the MIC. Gram-positive bacteria, including two strains of *Listeria monocytogenes* and three strains of *S. aureus,* also demonstrated a good sensitivity at low LLEO concentrations, with MIC values from 2.5 to 5 μL/mL. For these two species, the MMCs were equal to the MIC, except for the strains of *L. monocytogenes* DSM 15675 and *S. aureus* DSM 2569, where it doubles.

Our results evidenced that the antimicrobial activity against bacterial species tested were strain-dependent. Gram-negative bacteria were the most resistant and exhibited MICs from 10 to over 20 μL/mL. The MMCs were higher at the maximum concentration used. *S. bongori* and *E. coli* displayed a resistance to LLEO, highlighting that Gram-positive bacteria were more susceptible to Citrus EOs than Gram-negative bacteria.

### 3.3. Antioxidant Activity (DPPH Assay)

The radical scavenging abilities of LLEO were evaluated using the DPPH assay at 50 min. The antioxidant activity is given as IC_50_ value, which indicates the LLEO concentration required to give a 50% inhibition of the DPPH^•^ radical formation (Table 3). Values of IC_50_ determined in previous studies in similar experimental conditions for *C. limon* leaf, flower, and peel EO are also reported in Table 3 for comparison.

LLEO exhibited good antioxidant activity, with an IC_50_ value of 10.24 ± 2.8 mg/mL. However, the LLEO antioxidant activity was lower than the Trolox used as a reference antioxidant compound (IC_50_ value = 0.0069 mg/mL).

The radical scavenging activity of LLEO in DPPH assay was comparable to that previously reported for the EOs obtained by hydrodistillation from the leaves (IC_50_ value = 11.9 mg/mL) and peel (IC_50_ value = 12.9 mg/mL) of *C. limon* var. *pompia* grown in Sardinia [4,11], and for the EO obtained by hydrodistillation of leaves of *C. limon* cv. Femminello Comune from Rocca Imperiale (Italy) (IC_50_ value = 6.47 mg/mL) [18].

### 3.4. Cytotoxic Activity in Cancer and Normal Cells

The cytotoxicity of LLEO was investigated by the MTT colorimetric assay in different cancer and normal cell lines.

**Figure 3:**
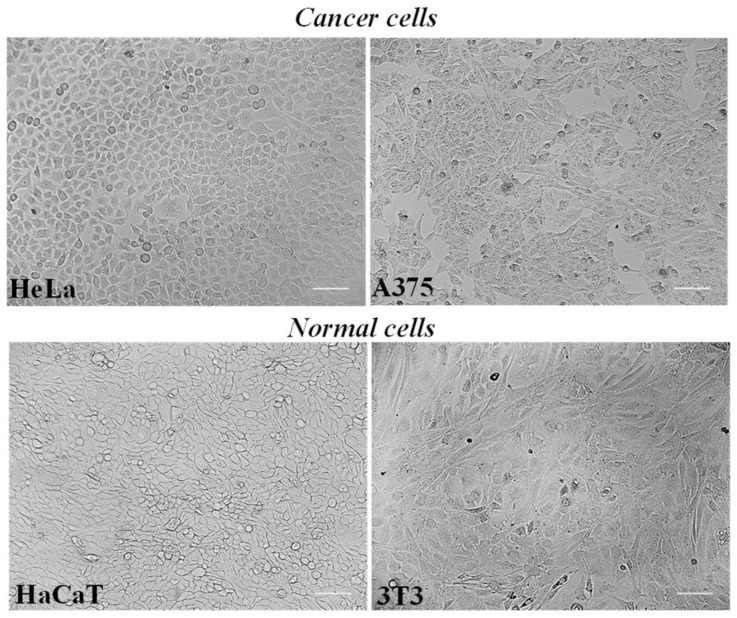
The panel shows representative images of phase contrast of human cancer HeLa cells, A375 human melanoma cells, human HaCaT keratinocytes, and 3T3 normal murine fibroblasts. Bar = 100 μm.

HeLa cells, a cell line derived from a human epithelioid cervix carcinoma, and A375 human melanoma cells represent cultured cancer cell models (Figure 3) amply used to assess the cytotoxic effect and potential antitumor properties of natural extracts and compounds [4,23,27,34,37].

Mouse 3T3 fibroblasts and human keratinocyte HaCaT cells were chosen as normal cell lines (Figure 3) as previously used to assess the biocompatibility of herbal extracts and natural-derived compounds [35,37].

**Figure 4:**
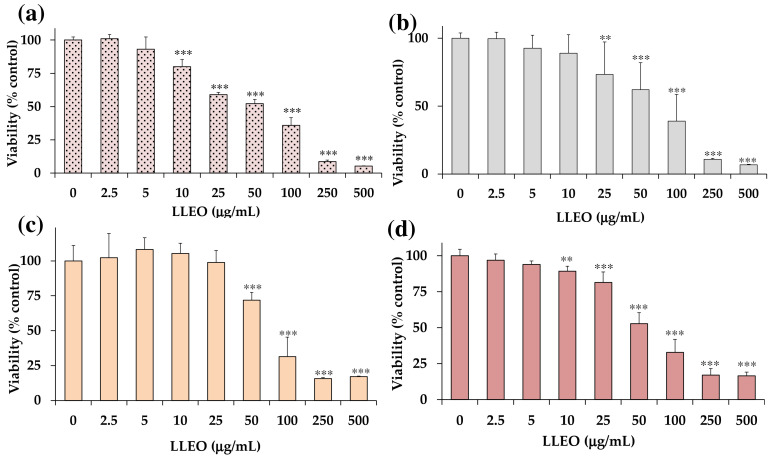
Viability, expressed as % of the control (0), induced by incubation for 24 h with different amounts (2.5–500 μg/mL) of the essential oil obtained from *C. limon* leaves (LLEO) in human cancer HeLa cells (**a**), A375 human melanoma cells (**b**), healthy human HaCaT keratinocytes (**c**), and 3T3 normal murine fibroblasts (**d**) (MTT assay). Three independent experiments are performed, and data are presented as mean and SD (n = 15). *** = *p* < 0.001, ** = *p* < 0.01 versus respective controls (0) (One-way ANOVA and Bonferroni post Test).

Figure 4 shows the viability, expressed as % of the control (0), induced by incubation for 24 h with different amounts (2.5–500 μg/mL) of LLEO in human cancer HeLa cells (Figure 4a), A375 human melanoma cells (Figure 4b), healthy human HaCaT keratinocytes, (Figure 4c), and 3T3 normal murine fibroblasts (Figure 4d) by MTT assay.

LLEO exerted a significant (*p* < 0.01) reduction (13%) in HeLa cell viability (Figure 4a), in comparison with control (untreated) cells, from the dose of 10 μg/mL. A dose-dependent cancer cell growth inhibition of 33–64% was observed at the concentration range of 25–100 μg/mL, while a 92–95% viability reduction was observed at the highest tested doses (250 and 500 μg/mL). The IC_50_ value (the concentration that decreases the cell viability to 50%) of LLEO after 24 h incubation in cancer HeLa cells was 56.5 μg/mL.

**Figure 5:**
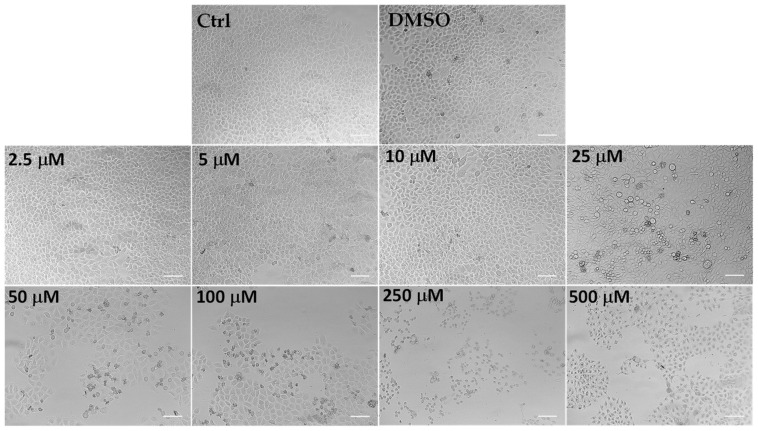
The panel shows representative images of phase contrast of control HeLa cells and cells treated for 24 h with *C. limon* leaf essential oil (LLEO) at 2.5–500 μg/mL. Bar = 100 μm.

Microscopic observation of HeLa cells treated for 24 h with LLEO (Figure 5), before the MTT assay, showed evidence of changes in cell morphologies with respect to control cells from the dose of 25 μg/mL.

Control (untreated) HeLa cells were small and closely linked to each other (packed), while the LLEO treatment induced, from 25 μg/mL, a reduction in the cell number and a remarkable increase in the number of cells with rounded morphology (apoptotic cells) in a concentration-dependent manner. Moreover, the occurrence of clear apoptotic bodies, cell blebbing and cell debris, loss of adhesion and cellular volume was observed from 25 μg/mL. At the highest LLEO concentration (500 μg/mL), unless a marked viability reduction was observed by MTT assay, differences in cell morphology/number were observed around 250 μg/mL, probably due to differences in the mechanism of toxicity.

DMSO, used to dissolve LLEO, was not toxic in HeLa cells, and at the maximal tested dose (1%), the cell viability was 91%, with cells showing the same morphological features as control cells (Figure 5).

An analogous treatment with LLEO in cancer A375 cells (Figure 4b) induced a significant (*p* < 0.01) viability reduction (27%) (Figure 4b), in comparison with control cells, from the dose of 25 μg/mL. Cancer cell viability reduction values of 38 and 61% were observed at 50 and 100 μg/mL, respectively, while an 89–93% decrease in cell viability was determined at the highest tested doses (250 and 500 μg/mL). The IC_50_ value in A375 cells after 24 h of incubation (76.2 μg/mL) was higher than the value determined in HeLa cells. The vehicle (DMSO) used for LLEO solution was not toxic (90% viability) at the maximal tested dose (1%). As observed in HeLa cells, LLEO treatment induced marked changes in A375 cancer cells from 25 μg/mL, including cell number reduction and increase in the rounded cells, apoptotic bodies, cell blebbing, and cell debris [34]

However, LLEO showed a lower cytotoxic effect in healthy human HaCaT keratinocytes (Figure 4c) than in cancer HeLa cells at all tested concentrations. The extract was not cytotoxic in the range of 2.5–25 μg/mL, inducing a significant cancer cell viability reduction of 38% at 50 μg/mL (*p* < 0.001 versus control cells) and a 69–84% viability inhibition (*p* < 0.001) at the concentration range of 100–500 μg/mL. DMSO, used to dissolve LLEO, was not toxic in HaCaT cells, and at the maximal tested dose (1%), the cell viability was 91%. The IC_50_ value of LLEO after 24 h incubation in HaCaT keratinocytes was 77.0 μg/mL.

**Figure 6:**
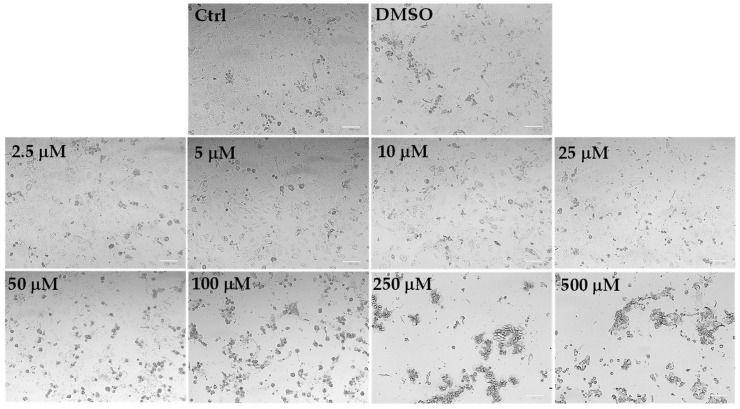
The panel shows representative images of phase contrast of control HaCaT keratinocytes and cells treated for 24 h with *C. limon* leaf essential oil (LLEO) at 2.5–500 μg/mL. Bar = 100 μm.

The microscopic observation, before the MTT assay, of HaCaT control cells and cells treated for 24 h with LLEO at the concentration range of 2.5–25 μg/mL evidenced similar morphological traits (spindle-shaped and adherent cells) as those observed for control (untreated) cells (Figure 6).

The addition of LLEO induced evident dose-dependent changes in HaCaT cell morphology with respect to control cells from the concentration of 50 μg/mL, as there was a remarkable increase in the number of rounded cells (apoptotic cells) and the occurrence of cell debris, shrunken and floating cells, signs indicative of cytotoxicity and cell death. Vehicle-treated cells (DMSO) showed the same morphological traits as control cells. These findings support the data recorded in the viability assay.

LLEO showed a lower cytotoxic effect in normal 3T3 fibroblasts (Figure 4d) than in cancer HeLa cells. No marked changes in cell viability, with respect to control cells, were observed in 3T3 fibroblasts treated with LLEO at 2.5 and 5 μg/mL. A significant low viability reduction, compared to controls, ranging from 11% (*p* < 0.01) to 19% (*p* < 0.001), was observed for LLEO in 3T3 cells at the dose of 10 μM and 25 μg/mL, respectively. A dose-dependent cancer cell growth inhibition of 47–83% was observed at the concentration range of 50–500 μg/mL. The IC_50_ value of LLEO after 24 h incubation in 3T3 fibroblasts was 57.5 μg/mL. The amount of DMSO used to dissolve the extract was not in a toxic range for 3T3 fibroblasts, and the cell viabilities, measured at the maximal tested dose (1%), were 90%. Changes in fibroblast cell morphology, with respect to control cells, after LLEO treatment were strictly similar to those observed in HaCaT cells [34].

A cell viability reduction was also observed in normal 3T3 fibroblasts and HaCaT keratinocytes after 24 h incubation with LLEO. However, a significantly less marked cytotoxic effect (MTT assay) versus cancer HeLa cells was observed for LLEO in normal 3T3 cells at 25 μg/mL (*p* < 0.001) and from 5 μg/mL (*p* < 0.001) (except at 100 μM) in HaCaT cells, indicating more selective toxicity towards malignant cells than normal cells.

### 3.5. Pro-oxidant Activity in Cancer HeLa Cells

The encountered beneficial effects of Citrus EOs in cancer cells have been partially correlated to their pro-oxidant effects on the cellular level [4].

Therefore, changes in the redox status in response to LLEO incubation were measured in cancer HeLa cells to evidence whether the reduced cell viability resulted from increased ROS inside cancer cells.

HeLa cells were incubated for 2 h with different concentrations of LLEO (2.5–500 μg/mL) and then the intracellular ROS generation during LLEO treatment was monitored by the H_2_-DCF-DA assay.

**Figure 7:**
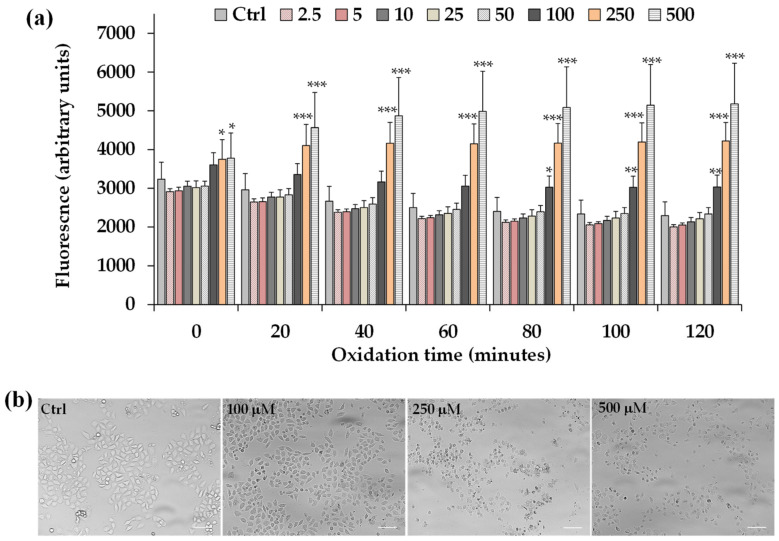
(**a**) ROS-induced fluorescence (arbitrary units), by H_2_-DCF-DA assay, measured at 0, 20, 40, 60, 80, 100, and 120 min in HeLa control cells (Ctrl) and cells exposed for 2 h to different amounts of *C. limon* leaf essential oil (LLEO) (from 2.5 to 500 μg/mL). Data were presented as mean ± SD (n = 9); *** = *p* < 0.001, ** = *p* < 0.01, * = *p* < 0.05 versus the Ctrl at each time point. Evaluation of the statistical significance of differences between groups was performed by one-way ANOVA followed by the Bonferroni Multiple Comparisons Test. (**b**) The panel shows representative images of phase contrast of control HeLa cells and cells after 2 h incubation with LLEO at 100, 250, and 500 μg/mL. Bar = 100 μm.

Figure 7a shows the time-dependent intracellular ROS generation measured during 2 h of incubation in control HeLa cells and cells treated with LLEO (2.5–500 μg/mL), while the panel of Figure 7b reports the representative images of phase contrast of control cells and cells after 2 h treatment with LLEO at 100, 250, and 500 μg/mL.

LLEO treatment induced a significant increase in the cell fluorescence during 2 h of incubation from the dose of 100 μg/mL compared to the basal rate of control cells, and the ROS generation was more marked at the LLEO highest tested dose (500 μg/mL). DMSO used to dissolve the extract did not affect ROS generation with respect to control cells.

The treatment for 2 h with LLEO induced, together with alterations in intracellular redox potential, marked changes in the cancer HeLa cell morphology and number in comparison with untreated cells, from the concentration of 100 μg/mL. Some morphological alterations were also observed after 2 h of incubation at the LLEO doses of 25 and 50 μg/mL without an evident cell fluorescence increase.

Our results evidenced that the LLEO cytotoxic activity in cancer cells could be partly related to the LLEO-induced ROS formation within cells.

### 3.6. Effect versus H_2_O_2_-Induced Oxidation in HaCaT Cells

Taking into consideration the observed antioxidant activity of LLEO in the DPPH assay, the potential protective effect of the extract was then explored in a cell-based system, against the oxidative stress induced in HaCaT cells by the treatment for 1 h with the oxidant compound H_2_O_2_. The H_2_O_2_-induced HaCaT cell damage model has been extensively researched regarding antioxidant effects [38].

HaCaT keratinocytes were pre-incubated for 24 h with a non-toxic concentration (10 μg/mL) of LLEO and the increase in the intracellular ROS level was determined after H_2_O_2_ exposure by the H_2_-DCF-DA assay. This concentration was chosen to evidence the eventual protective effect of LLEO effects on H_2_O_2_-induced ROS generation at a very low level of cell mortality (11%) and low compromise of cell functionality.

**Figure 8:**
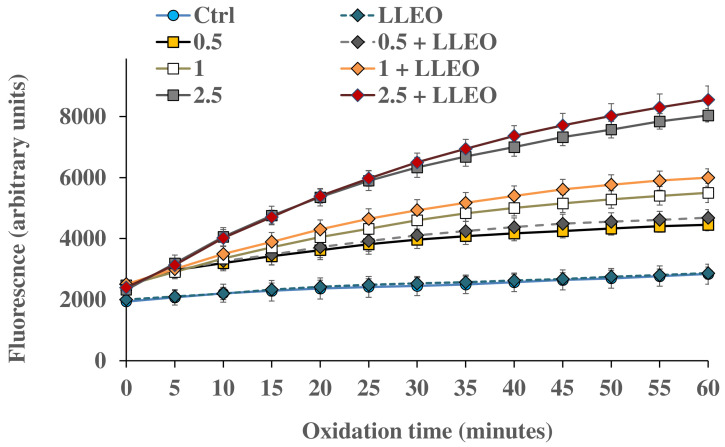
ROS-induced fluorescence (arbitrary units), by H_2_-DCF-DA assay, measured at different time points (every 5 min) in HaCaT control cells (Ctrl) and cells exposed for 1 h to different amounts of H_2_O_2_ (0.5, 1, and 2.5 mM) in the absence and in the presence (24 h of pre-incubation) of *C. limon* leaf essential oil (LLEO) at the dose of 10 μg/mL (0.5 + LLEO, 1 + LLEO, 2.5 + LLEO). Data were presented as mean ± SD (n = 9). At each time point, significant differences (*p* < 0.001) were observed for all oxidized samples (in the absence and in the presence of LLEO) versus the respective Ctrl. Evaluation of the statistical significance of differences between groups was performed by one-way ANOVA followed by the Bonferroni Multiple Comparisons Test.

Figure 8 shows the intracellular ROS generation measured at different time points in control HaCaT cells and cells exposed for 1 h to H_2_O_2_ (0.5, 1, and 2.5 mM) in the absence and in the presence (24 h of pre-incubation) of LLEO (10 μg/mL).

The treatment for 1 h with H_2_O_2_ induced a significant increase in cell fluorescence during 60 min time of exposure compared to the basal rate of control cells, and the highest relative intensity of fluorescence in HaCaT cells was observed at the highest oxidant concentration (2.5 mM).

HaCaT cells preincubated for 24 h with LLEO at 10 μg/mL showed the same basal ROS level as control cells, indicating the absence of pro-oxidant properties. However, in our experimental conditions, LLEO pretreatment did not exert protection against ROS generation induced by H_2_O_2_.

A slightly higher ROS level (unless not significant) was observed for HaCaT cells pretreated with LLEO in comparison with H_2_O_2_-oxidized cells, highlighting a certain pro-oxidant effect of the extract in the presence of the oxidant (at 1 and 2.5 mM of H_2_O_2_).

At 10 μg/mL, LLEO did not directly induce changes in intracellular redox potential and toxic effects (as observed by MTT assay and morphological observation); however, it probably made cultured cells more sensitive to an external oxidant.

## 4. Discussion

In this work, the pleiotropic activity of LLEO was examined in relation to the chemical compositions.

LLEO was extracted by hydrodistillation from lemon leaves with a high yield, amounting to over 2% *v*/*w*. The EO content of *C. limon* leaves obtained in the present analysis was found to be higher than that previously reported for the EO obtained by hydrodistillation from the leaves of *C. limon* var. *pompia* grown in Sardinia, characterized by a yield ranging between 0.43% (*v*/*w*) and 0.52% (*v*/*w*) calculated from the dry weight [11]. Yield values of 0.41% and 0.56%, expressed on a fresh weight basis (*v*/*w*), were reported for the EO obtained by hydrodistillation from the leaves of *C. limon* plants collected in the South of Iran [15] and Nubaria district (Egypt) [17], respectively, whereas yields of 0.338% and 0.33% (*w*/*w*) (calculated based on the initial plant weight) were determined for EOs prepared by traditional hydrodistillation of discarded leaves of *C. limon* cultivated in China [16] and Tunisia [10], respectively. The yield, expressed as mL of EO per kg of plant material fresh weight, of leaf EO obtained from a Cretan lemon variety varied from 3.5 to 5.3 mL/kg [39]. The observed differences in lemon leaf EO yields are strictly correlated with the plant variety and/or cultivars, the geographic origin, and the harvesting period [3,13,15,39].

GC-MS analysis revealed limonene as the major component followed by citral, as the sum of geranial and neral. In qualitative terms, the chemical composition of the LLEO analyzed in this work was very similar to that of EO previously obtained by hydrodistillation from the leaves of *C. limon* var. *pompia* grown in Sardinia, characterized by limonene (256 mg/mL), geranial (213.8 mg/mL), and neral (172.9 mg/mL) as the major compounds [11]. Moreover, our results agreed with those previously reported for leaf EO extracted from lemon plants of various origins, characterized by high concentrations of limonene [10,13,18,39,40]. EO obtained by hydrodistillation of leaves of *C. limon* cv. Femminello Comune from Rocca Imperiale (Italy) revealed the presence of 36 main constituents and the most abundant compound was limonene (27.58%), followed by beta-pinene (17.10%), geranial (7.40%), neral (6.67%), and sabinene (5.10%) [18]. The EO of leaves from the Cretan variety Zambetakis of *C. limon*, obtained by steam distillation with a Clevenger apparatus, also showed limonene as the main component, followed by alpha-pinene, myrcene, neral, geranial, neryl acetate, geranyl acetate, and alpha-caryophyllene [39]. Interestingly, the leaf EO of *C. limon* (L.) Osbeck growing in southwestern Nigeria was also rich in limonene (31.5%), sabinene (15.9%), citronellal (11.6%), linalool (4.6%), neral (4.5%), and geranial (4.5%) [40], whereas the hydrodistilled EO of *C. limon* leaves cultivated in Tunisia contained geranial (30.08%), limonene (27.09%), and neral (22.87%) as the predominant compounds in the identified peaks [10].

In some cases, other compounds have been identified as the major constituents [10,15,16,17,19]. Linalool (30.6%) was identified as the main compound in the leaf EO of lemon cultivated in the South of Iran [15]. A study conducted on several taxa of lemons cultivated on the island of Corsica (France) in the same pedoclimatic and cultural conditions allowed the identification of two chemotypes for lemon leaf EO: limonene/beta-pinene/geranial/neral and linalool/linalyl acetate/α-terpineol [41]. Furthermore, citronellal and sabinene have been reported as the most abundant components in the leaf EO obtained from lemons cultivated in China [16] and Egypt [17], respectively. It is well-known that different factors, including genetic, geographic, and seasonal variation, as well as the cultivar, species, ripening stage, cultural practices, extraction methods, and environmental/climate conditions greatly influence the qualitative and quantitative chemical composition and yield of leaf EOs [3,13,15].

There is a growing demand and attention of industry and consumers in the use of herbal extracts and naturally derived compounds as ingredients/additives, an alternative to synthetic ones, to prevent the proliferation of microorganisms during the production, sale, and distribution of food products and to extend the shelf life of raw and/or processed foods [1]. Citrus plants constitute one of the main sources of EOs extensively studied for their potential uses as antimicrobial preservative agents in the food industry [1,3,9].

LLEO demonstrated a good inhibitory activity against the pathogenic microorganisms *C. albicans*, *L. monocytogenes,* and *S. aureus*. The LLEO antimicrobial activity against tested bacterial species was strain dependent [11,12] and Gram-positive bacteria were more susceptible to lemon EO than Gram-negative bacteria were, as previously shown by other authors [3,11,15].

Our results are in line with those previously reported for leaf EO extracted from lemons of various origins. Studies previously conducted on the antimicrobial activities of the EO obtained by hydrodistillation from the leaves of *C. limon* var. *pompia* [11], rich in limonene, geranial, and neral, demonstrated its ability to inhibit *L. monocytogenes* and *S. aureus* at low EO concentration (MIC = 2.5 μL/mL). *C. limon* var. *pompia* leaf EO also demonstrated antimicrobial activity against yeast, with *S. cerevisiae* being the most sensitive strain [11]. The potent inhibitory activity of *C. limon* var. *pompia* leaf EO has been ascribed to the high concentration of oxygenated compounds (58.5%) [11].

The oil obtained from *C. lemon* leaves as pruning materials collected in Nubaria district (Egypt), characterized by sabinene, carene, limonene, and β-ocimene as main components, showed a remarkable inhibition against *S. aureus* (MIC = 0.2 μL/mL) and *P. aeruginosa* (MIC = 0.4 μL/mL) determined by optical density assay, with a strong effect on the DNA, RNA, lipids, and protein biosynthesis in cells of *S. aureus* and on the biosynthesis of the lipids in cells of *P. aeruginosa* [17].

The leaf EO obtained from *C. limon* cultivated in the South of Iran, rich in linalool, geraniol, alpha-terpineol, and linalyl acetate, showed in the range 0.2–10 mg/mL a significant microbial activity against the Gram-positive bacteria *Streptococcus faecium*, *Bacillus cereus*, and *S. aureus* [15].

Natural EOs have been viewed as potential candidates to combat antimicrobial resistance due to their complex chemistry, which carries inherent pro-oxidant and antioxidant properties [42]. Citrus EOs have been confirmed as an alternative to synthetic antimicrobials [9] and their activity at the cellular level consists of many modes of action [9]. Low-molecular-weight compounds of EOs allow them to easily penetrate through cell walls, affect various biochemical processes, and induce irreversible damage of bacterial membranes, resulting in cytoplasmic losses, energy substrate loss causing bacterial lysis, ion leakage, and death [9,15,17]. Another possible action mode is protease inhibition and therefore cell content coagulation [17].

The research of new antioxidants is a hot topic, especially in the field of natural products [18]. The use of EOs as natural antioxidants is a field of growing interest, especially in food science and in complementary medicine [43]. Several Citrus EOs have shown remarkable antioxidant properties in biological systems and foodstuffs [4,8,9,11,13]. LLEO activity in DPPH assay was comparable to that previously reported for the EOs obtained by hydrodistillation from the leaves (IC_50_ value = 11.9 mg/mL; limonene, geranial, and neral as main components) and peel (IC_50_ value = 12.9 mg/mL, characterized by high amount of limonene) of *C. limon* var. *pompia* grown in Sardinia [4,11].

An IC_50_ value of 6.47 mg/mL was previously reported for the EO obtained by hydrodistillation of leaves of *C. limon* cv. Femminello Comune from Rocca Imperiale (Italy), characterized by limonene, beta-pinene, geranial, and neral as main constituents [18]. The distilled leaf EO obtained from a commercially cultivated *C. limon* in Malaysia, characterized by limonene as the major compound, showed an IC_50_ value of 29.14 mg/mL [27]. Hojjati and Barzegar [15] reported an IC_50_ value of 0.98 mg/mL for the leaf EO of lemon cultivated in the South of Iran, characterized by linalool as the main compound, followed by geraniol, alpha-terpineol, and linalyl acetate.

LLEO showed a certain degree of radical scavenging activity in the DPPH assay, as previously observed for other Citrus EOs. The antioxidant potency of an EO strictly depends on its composition, experimental conditions, and oxidizable material; in general, EOs having a high content of phenols, and cyclohexadiene-like components are more active in in vitro systems of oxidative stress [43]. Differences observed in the antioxidant potential of leaf EOs extracted by hydrodistillation from lemon plants of various origins might be attributed to the variations in their phytoconstituents [27].

A previous study on the evaluation of DPPH radical scavenging activity of *C. limon* cv. Eureka and cv. Lisbon peel EO and their main identified constituents revealed that geraniol, terpinolene, and gamma-terpinene were the most active against DPPH radicals [44]. Few studies have reported that the antioxidant activity of Citrus EOs might be correlated to the level or proportion of limonene, with higher antioxidant activity in oils containing a higher proportion of limonene [18,27]. DPPH radical scavenging activity of EO from *C. limon* (L.) BURM. cv. Femminello Comune was positively correlated with monoterpene hydrocarbons and the main abundant compounds (limonene, gamma-terpinene, and beta-pinene) [18].

Strong evidence indicates that Citrus EOs have remarkable anticancer effects on the growth, invasion, angiogenesis, and metastasis of different tumor cells and have been proposed as a promising agent for cancer therapy [9]. The cytotoxicity of LLEO was investigated by MTT assay in cancer HeLa cells and A375 human melanoma cells. We previously used human epithelioid cervix carcinoma HeLa cells to assess the cytotoxic and/or proapoptotic properties of natural extracts/compounds [4,34]. Human malignant melanoma A375 cells, derived from a primary skin melanoma with an epithelioid morphology, are one of the most frequently used melanoma cell lines for research studies [37]. LLEO showed, after 24 h of incubation, marked cytotoxic activity on both cancer cell lines, greatly affecting cancer cell morphology.

Several investigations have shown the growth inhibitory effect and antiproliferative activity of *C. limon* EOs from different plant parts and origins and their chemical constituents as antitumor agents in various cancer cell lines [4,27,28,45,46]. A potent dose-dependent antiproliferative activity against cancer HeLa cervical cells, with an IC_50_ value of 11.66 μg/mL at 24 h of incubation, was previously observed for steam-distilled leaf EO obtained from commercially *C. limon* cultivated in Malaysia, characterized by limonene (33.6%) and citral (33.6%) as major compounds [27]. The leaf EO, extracted by hydrodistillation method from *C. limon* cultivated in India [28], rich in alpha-pinene, beta-pinene, decanal, citral, and alpha-terpineol, presented a much higher cytotoxicity (IC_50_ = 4.75 μg/mL) in cancer HeLa cells compared to the current study.

Interestingly, the in vitro cytotoxicity activity of *C. limon* peel oil from Northern Egypt (56% of limonene) against the HeLa cell line resulted in an IC_50_ value of 51.0 μg/mL [45]. *C. limon* var. *pompia* peel EO, containing 803.8 mg/mL limonene (accounting for 90% of the whole composition), exhibited cytotoxicity against cancer HeLa cells and B16F10 melanoma with an IC_50_ value of 408.0 and 148 μg/mL, respectively [4]. EO obtained by hydrodistillation in a Clevenger apparatus from the peel of *C. limon* collected from the YanCheng area (China) showed an inhibition rate of HeLa cell growth of 39% at 60 μg/mL EO concentration [23]. The EO distilled from the peels of *C. limon* fruits collected in Iran, rich in limonene (98.4%), exhibited an IC_50_ value of 17 μg/mL in HeLa cells [46].

GC-MS analysis revealed limonene as the major component in LLEO, followed by citral (the oxygenated monoterpenes geranial and neral). The cytotoxicity of Citrus EOs has been largely ascribed to their main component limonene, a non-phenolic terpenoid, well-established as a chemopreventive and therapeutic agent against numerous tumor cells (MCF-7, MGC803, K562, A-549, PC 12, HT-29, HeLa cell lines, and HepG2 hepatocarcinoma cells) [4,9,13,21,25,26,47]. In particular, D-limonene induced apoptosis in LS174T human colon cancer cells via the mitochondrial death pathway and the suppression of the PI3K/Akt pathway [13]. Moreover, a previous study evidenced the ability of citral to suppress cell proliferation, through the increase in intracellular ROS and dissipation of mitochondrial membrane potential in HeLa cells [27]. Citral also showed cytotoxic effects and induction of apoptosis in several cancer cell lines [21,25,26]. Other compounds with a cytotoxicity effect against various cancer cell lines include myrcene and linalool [25,26].

These findings suggest that different constituents in LLEO may synergistically contribute to their antiproliferative activity against cancer HeLa and A375 cells, instead of being the sole contribution of a single bioactive compound.

The potent anticancer activity of EOs and their constituents are the results of multiple pathways and mechanisms involving apoptosis, cell cycle arrest, antimetastatic/antiangiogenic activities, increased levels of reactive radical species, DNA repair modulation, loss of key organelle function, and effects on tumor suppressor proteins, transcription factors, and detoxification enzymes [4,21,25,37,47]. Due to their lipophilic nature and low molecular weights, EO constituents can cross cell membranes, increasing membrane fluidity, altering the phospholipid layers, and leading to leakage of ions/radicals and cytoplasmic content [4,21,25,37,47]. In particular, the efficiency of EOs and their constituents in reducing tumor cell proliferation has been correlated to their pro-oxidant effect [4,21].

A cell viability reduction (MTT assay) was also observed in normal 3T3 fibroblasts and HaCaT keratinocytes after 24 h incubation with LLEO. However, a significantly less marked cytotoxic effect versus cancer HeLa cells was observed for LLEO in normal 3T3 cells at 25 μg/mL (*p* < 0.001) and from 5 μg/mL (*p* < 0.001) (except at 100 μM) in HaCaT cells, indicating more selective toxicity towards malignant cells than normal cells.

A previous study showed the cytotoxic effect against normal murine macrophages (RAW 264.7 cell line), with an IC_80_ value of 3.32 ± 0.24 μg/mL, of the hydrodistilled EO of *C. limon* cultivated in Tunisia, containing geranial, limonene, and neral as the predominant compounds [10]. Moreover, a toxic effect of the terpenes limonene (IC_50_ value = 1.58 mM) and alpha-terpineol (IC_50_ value = 130 μM) was reported in Balb/c 3T3-A31 fibroblasts after 48 h exposure and correlated to their ability to increase cell membrane fluidity [48].

Many radical-producing agents are used in antitumor treatments, and the pro-oxidant effects of EOs in cancer cells are related to their interference with mitochondrial functions [4]. The increase in ROS production is the most frequently encountered phenomenon in cancer cells in response to the EO treatment that leads to cell death by induction of apoptosis [4,47]. Furthermore, the Citrus EOs’ beneficial effects in cancer cells have been partially correlated to their pro-oxidant effects on the cellular level [4].

The treatment for 2 h with LLEO induced a marked increase in HeLa cells’ intracellular redox potential, evidencing that the LLEO cytotoxic activity in cancer cells could be partly related to the LLEO-induced ROS formation within cells. We previously demonstrated that the toxic effect of the C. limon var. pompia peel EO in cancer murine melanoma B16F10 cells was partly achieved by ROS generation inside cancer cells [4].

In vitro physicochemical assays characterize most of EOs as antioxidants; however, they can act as pro-oxidants in eukaryotic living cells, affecting inner cell membranes and inducing changes in intracellular redox potential and mitochondrial dysfunction [29,47]. The cytotoxic effect of EOs in living cells has been related to their ability to affect the cellular redox status, acting as pro-oxidants [29,47].

The LLEO effect was explored in normal HaCaT cells against the oxidative stress induced by the 1 h treatment with the oxidant H_2_O_2_. H_2_O_2_ can not only cause lipid peroxidation to destroy cell integrity but also induce apoptosis by disrupting mitochondria [38]. In our experimental conditions, LLEO did not directly induce changes in intracellular redox potential; however, it probably made cultured cells more sensitive to the external oxidant.

## 5. Conclusions

The chemical composition and biological properties of EO extracted from the leaves of *C. limon* were investigated. The leaves used for the extraction were obtained by the pruning of lemon trees, biomass often discarded as an agro-industrial waste product. LLEO, rich in limonene (260.7 mg/mL) and citral (194.5 mg/mL), showed DPPH radical scavenging activity (IC_50_ value = 10.24 mg/mL) and demonstrated a good inhibitory activity against the pathogenic microorganisms *C. albicans* (MIC = 0.625 μL/mL) *L. monocytogenes* and *S. aureus* (MIC values from 2.5 to 5 μL/mL). Moreover, the treatment with LLEO significantly affected cell viability and morphology from 25 μM in cancer HeLa and A375 cells, with lower effects on normal fibroblasts and keratinocytes. Moreover, we demonstrated that the LLEO ability to affect microbial and cancer cell viability could probably be related to its capacity to affect the cellular redox status by the generation of ROS inside cells and cell membrane fluidity.

Taking into consideration the pleiotropic bioactivity of LLEO, its high yield (amounting to over 2% *v*/*w*), and the waste origin of the extracted material, the results of our research provided a useful approach/information for the valorization of discarded lemon leaves as a source of bioactive EO with potential application in food, cosmetic, and pharmaceutical industries, from the perspective of circular and green economy chemistry. However, the use of LLEO for pharmaceutical/cosmeceutical applications and as food preservatives will require further toxicological assessment and the evaluation of the safety range. The possibility of mass implementation of LLEO production for food and cosmetic application in the future could be economically and ecologically viable.

## Figures and Tables

**Table 1:** Chemical composition (expressed as mg/mL of extract) by GC-FID of EO obtained from the leaves of *Citrus limon* (LLEO), the retention index (RI), retention index from the literature (RI_Lit_), calibration curves, and R^2^ values.

Compound	RI	RI_Lit_	mg/mL	Calibration Curve	R^2^
Myrcene	986.0	991	94.7 ± 1.9	Limonene	
3-Carene	1005.6	1007	20.6 ± 1.2	Limonene	
Limonene	1024.9	1028	260.7 ± 2.4	y = 169.87x + 35.588	0.9979
beta-Ocimene	1045.1	1050	15.5 ± 0.4	Limonene	
gamma-Terpinene	1053.2	1056	10.9 ±1.2	Limonene	
Linalool	1100.6	1101	24.5 ± 0.3	Neral	
6-Octenal,7-methyl-3-methylene	1145.3	1146	9.6 ±0.8	Limonene	
Citronellal	1151.6	1153	15.3 ± 0.1	Neral	
Isoneral	1164.7	1165	10.1 ± 0.2	Neral	
Terpinen-4-ol	1172.0	1172	11.7 ± 0.5	Neral	
Isogeranial	1182.7	1184	11.3 ± 0.5	Geranial	
alpha-Terpineol	1187.9	1189	15.0 ± 0.2	Neral	
Nerol	1233.6	1226	14.2 ± 0.1	Neral	
Citronellol	1236.2	1228	Tr	Neral	
Neral	1244.7	1240	88.3 ± 1.3	y = 179.29x + 134.07	0.9961
Geraniol	1261.2	1254	14.0 ± 0.9	Geranial	
Geranial	1275.7	1271	106.2 ± 1.6	y = 193.32x + 57.631	0.9997
Citronellyl-propanoate	1355.8	1444	Tr	Neral	
Neryl acetate	1366.9	1362	31.8 ± 0.9	Neral	
Geranyl acetate	1385.0	1382	23.5 ± 0.9	Geranial	
Z-Caryophyllene	1405.6	1409	9.0 ± 0.5	y = 223.94x − 33.917	0.9983

Legend: RI = retention index determined on an HP-5 capillary column relative to a series of n-alkanes; RI_Litt_ = retention index reported from NIST libraries (available online); Tr = compounds present in trace.

**Table 2:** Minimum inhibitory concentrations (MICs) and minimum microbicidal concentrations (MMCs), expressed as μl/mL, of the essential oil obtained from *C. limon* leaves (LLEO) against the tested microorganisms determined after 24 h of incubation.

Tested Organisms	MIC	MMC
*Candida albicans* 3933	0.625	1.25
*Candida albicans* 3993	0.625	1.25
*Staphylococcus aureus* DSM 20231	2.5	2.5
*Staphylococcus aureus* DSM 2569	2.5	5
*Staphylococcus aureus* DSM 6148	2.5	2.5
*Listeria monocytogenes* DSM 20600	2.5	5
*Listeria monocytogenes* DSM 15675	5	10
*Escherichia coli* DSM 30083	10	10
*Escherichia coli* DSM 4415	20	>20
*Salmonella bongori* DSM 13772	>20	>20

**Table 3:** Antiradical effects (DPPH assay) of the essential oil obtained from *C. limon* leaves (LLEO). The antioxidant activity of other distilled *C. limon* leaf, flower, and peel EO obtained in similar experimental conditions is reported for comparison.

Antioxidant	IC_50_ (mg/mL) ^a^	Oxidation Time	Literature Reference
LLEO	10.24 ± 2.8	50 min	-
Trolox	0.00689 ± 0.00040	50 min	-
*C. limon* var. *pompia* leaf EO	11.9	60 min	[11]
*C. limon* L. leaf EO	29.14 ± 1.97	20 min	[27]
*C. limon (L.)* Burm. cv. Femminello Comune leaf EO	6.47 ± 0.1	30 min	[18]
*C. limon* leaf EO	0.98	60 min	[15]
*C. limon* flowers EO	0.015	20 min	[1]
*C. limon* var. *pompia* peel EO	12.9	60 min	[4]
*C. limon (L.)* Burm. cv. Femminello Comune peel EO	1.17 ± 0.06	30 min	[18]

^a^ IC_50_ value: indicates the LLEO concentration required to give a 50% inhibition of the DPPH^•^ radical formation.

## Data Availability

The datasets generated and analyzed during the current study are available from the corresponding author on reasonable request.
